# Impaired Corpus Cavernosum Relaxation Is Accompanied by Increased Oxidative Stress and Up-Regulation of the Rho-Kinase Pathway in Diabetic (Db/Db) Mice

**DOI:** 10.1371/journal.pone.0156030

**Published:** 2016-05-26

**Authors:** Fernanda B. M. Priviero, Haroldo A. F. Toque, Kenia Pedrosa Nunes, Denise G. Priolli, Cleber E. Teixeira, R. Clinton Webb

**Affiliations:** 1 Laboratory of Multidisciplinary Research, Universidade São Francisco, Bragança Paulista, São Paulo, Brazil; 2 Department of Pharmacology and Toxicology, Augusta University, Augusta, Georgia, United States of America; 3 Department of Biological Sciences, Florida Institute of Technology, Melbourne, Florida, United States of America; 4 Department of Physiology, Augusta University, Augusta, Georgia, United States of America; University of Illinois at Chicago, UNITED STATES

## Abstract

Basal release of nitric oxide from endothelial cells modulates contractile activity in the corpus cavernosum via inhibition of the RhoA/Rho-kinase signaling pathway. We aimed to investigate nitric oxide bioavailability, oxidative stress and the Rho-kinase pathway in the relaxation of the corpus cavernosum of an obese and diabetic model of mice (db/db mice). We hypothesized that in db/db mice impaired relaxation induced by Rho-kinase inhibitor is accompanied by diminished NO bioavailability, increased oxidative stress and upregulation of the RhoA/Rho-kinase signalling pathway. Cavernosal strips from male lean and non-diabetic db/+ and db/db mice were mounted in myographs and isometric force in response to Rho-kinase inhibitor Y-27632 was recorded. Enzyme activity and protein expression of oxidative stress markers and key molecules of the RhoA/Rho-kinase pathway were analyzed. The Rho-kinase inhibitor Y-27632 concentration-dependently caused corpus cavernosum relaxation and inhibited cavernosal contractions. Nonetheless, a rightward shift in the curves obtained in corpus cavernosum of db/db mice was observed. Compared to db/+, this strain presented increased active RhoA, higher MYPT-1 phosphorylation stimulated by phenylephrine, and increased expression of ROKα and Rho-GEFs. Further, we observed normal expression of endothelial and neuronal NOS in corpus cavernosum of db/db mice. However, nitrate/nitrate (NOx) levels were diminished, suggesting decreased NO bioavailability. We measured the oxidant status and observed increased lipid peroxidation, with decreased SOD activity and expression. In conclusion, our data demonstrate that in db/db mice, upregulation of the RhoA/Rho-kinase signalling pathway was accompanied by decreased NO bioavailability and increased oxidative stress contributing to impaired relaxation of the corpus cavermosum of db/db mice.

## Introduction

Penile erection is achieved by cavernosal smooth muscle relaxation and nitric oxide (NO) has been considered the main mediator of this relaxation.[1] Nerve- and endothelium- released NO targets soluble guanylyl cyclase in the corpus cavernosum (CC), thus increasing cGMP levels, which activate protein kinase G (PKG). Activated PKG decreases intracellular levels of calcium causing smooth muscle relaxation. [1–4] The penis is kept in the flaccid state because of adrenergic activation, and in the absence of an active NO/cGMP pathway, the cavernosal smooth muscle remains in the contracted state. This is mediated by the effects of noradrenaline released from sympathetic nerves and prostaglandin F2α and endothelin from the endothelium, increasing intracellular calcium concentration. [1, 5, 6] In addition, the RhoA/Rho-kinase pathway plays an important role in maintenance of penile flaccidity. Its upregulation has been associated with erectile dysfunction. [7–9]

Erectile dysfunction is described as a persistent inability to achieve and/or maintain penile erection for satisfactory sexual intercourse and it is also associated with several disorders such as arterial hypertension, atherosclerosis, hypercholesterolemia, diabetes, obesity, metabolic syndrome, sleep apnea, cigarette smoking and aging. [10–18] Also, these disorders often diminish NO bioavailability. One of the main causes of reduced NO bioavailability is oxidative stress. Oxidative stress increases the level of reactive oxygen species (ROS), which react with NO and prevent the binding of NO to its target.

In the last decade, there have been a growing number of studies focused on oxidative stress and cardiovascular disease. ROS are continuously formed as a product of cellular metabolism. In the healthy state, there is a balance between ROS production and elimination, which is performed by enzymatic and non-enzymatic antioxidants agents such as SOD, catalase, peroxidase, and vitamins C and E. Oxidative stress occurs when the antioxidant capacity is diminished and/or ROS production is increased, causing an imbalance between ROS production and elimination in favour of their production. Beyond NO inactivation, ROS also binds to lipids and proteins, causing cellular damage through lipid and protein oxidation. [12, 19] ROS have been shown to be involved in several cardiovascular diseases, and is associated with increased NADPH oxidase expression and activity. [20]

We have previously demonstrated that the basal release of endothelial NO plays an essential role in the maintenance of erectile function, as its deficiency causes amplification of the RhoA/Rho-kinase pathway. [21] Additionally, we have recently shown increased NADPH oxidase and impaired NO-induced relaxation in db/db mice. [22] Thus, we hypothesized that RhoA/Rho-kinase signalling pathway might be upregulated in db/db mice. Herein, we found impaired relaxation induced by Rho-kinase inhibitor, which was accompanied by diminished NO bioavailability, increased oxidative stress and increased expression/activity of the proteins related to RhoA/Rho-kinase pathway.

## Methods

### Animals

All experimental procedures were conducted in accordance with institutional guidelines and approved by the Georgia Regents University Institutional Animal Care and Use Committee. Male C57BL/KsOlaHsd-lepr^db^/lepr^db^ mice (db/db, mice with obesity and type II diabetes caused by a leptin receptor mutation) and their lean, non-diabetic heterozygote (db/+) C57bl/6kso littermates (14–16 weeks old, Harlan, Indianapolis, IN, USA) were used in all experiments. Animals were housed in temperature-controlled facilities on a 12-hour light-dark cycle with *ad libitum* food and water access. Before sacrifice, arterial blood was collected from the abdominal aorta to NOx and 8-isoprostane levels and SOD activity measurements.

### Functional studies

The animals were stunned by inhalation of CO_2_, sacrificed by decapitation and exsanguinated. The penises were surgically removed and placed in chilled physiological buffer of the following composition (mmol/L): NaCl, 130; NaHCO_3_, 14.9; dextrose, 5.5; KCl, 4.7; KH_2_PO_4_, 1.18; MgSO_4_7H_2_O, 1.17 and CaCl_2_2H_2_O, 1.6. Following removal of the glans penis and urethra, the penile tissue was cleaned from connective and adventitial tissues and the fibrous septum separating the corpora cavernosa was opened from its proximal extremity towards the penile shaft. A slit was made in the tunica albuginea along the shaft to obtain two strips (11x1x1 mm) of corpus cavernosum (CC) from each animal. Each strip was mounted in a myograph for isometric force recording (Danish Myograph Technology, Aarhus, Denmark) coupled to a PowerLab 8/SP^™^ data acquisition system (software Chart 5.0, ADInstruments, Colorado Springs, U.S.A.). The bathing solution was maintained at 37°C and continuously aerated with 95% O_2_ and 5% CO_2_. Tissues were allowed to equilibrate for 45 min under a resting tension of 2 mN. Repetitive supramaximal transmural electrical field stimulation (EFS) of autonomic nerves was delivered via platinum pin electrodes placed on either side of the cavernosal strips. Electrodes were attached to a stimulus splitter unit (Stimu-Splitter II), which was connected to a Grass S88 stimulator (Astro-Med Industrial Park, U.S.A.).

After equilibration, the ability of the preparations to develop contraction was assessed in 80 mmol/L K^+^-substituted physiological buffer. Next, endothelial function was assessed by applying acetylcholine (ACh, 10 μmol/L) to strips contracted with phenylephrine (PE, 10 μmol/L). The relaxation induced by the Rho-kinase inhibitor Y-27632 (0.01–30 μmol/L) was obtained by cumulative concentration-response curves in PE-precontracted CC (10 μmol/L). In the other strip, EFS-induced contraction (16 Hz) was evaluated in the absence or in the presence of increasing concentrations of Y-27632 (0.01–30 μmol/L). EFS-induced contractions were calculated as a percentage of the maximal contraction induced by KCl (80 mmol/L) and were made in the presence of the NOS inhibitor L-NAME (100 μmol/L) and in the presence of the muscarinic receptor antagonist atropine (1 μmol/L). EFS (16 Hz) was applied in strips placed between two platinum ring electrodes connected to a Grass S88 stimulator (Astro-Med Industrial Park, RI, USA). EFS was conducted at 20 V, 1 ms pulse width and trains of stimuli lasting 10 sec for each frequency.

### Serum/Plasma, urine and tissue collection

Samples of urine and serum or plasma were collected and used for determination of NOx levels, 8-isoprostane levels and SOD activity. Homogenates of corpus cavernosum were used to measure 8-isoprostane levels and SOD activity.

Briefly, immediately after arterial blood collection (with or without EDTA as appropriate for plasma or serum samples, respectively), samples were centrifuged (1,000g) for 10 min at 4°C. The top yellow serum layer was collected without disturbing the white buffy layer and frozen at –80°C. Urine was collected through the insertion of a needle in the bladder and aspirated using a syringe. Samples were promptly frozen at −80°C. After excising and cleaning the penises as described in the section above, samples of corpus cavernosum were immediately frozen at −80°C. On the day of the experiment, tissue homogenates were processed as described in the instructions included in SOD and 8-iso-PGF2α assay kits. The assays were performed in duplicates using different sample dilutions.

### SOD activity determination

Prior to SOD activity determination, serum was diluted (1:5) with sample buffer. Tissue homogenate was obtained after maceration of the cleaned penis. Macerate was homogenized in 5–10 mL of cold HEPES buffer (20 mmol/L), containing EGTA (1 mmol/L), mannitol (210 mmol/L) and sucrose (70 mmol/L). After centrifugation (1,500 x g, five minutes, at 4°C), supernatant was collected and kept on ice for assay.

SOD activity was measured using a SOD Assay Kit (Cayman Chemical, Ann Arbor, MI) according to the manufacturer’s instructions. SOD activity is assessed by measuring the dismutation of superoxide radicals generated by xanthine oxidase and hypoxanthine.

### Determination of 8-isoprostane levels

Prior to determination of 8-isoprostane, macerate of corpus cavernosum was homogenized in homogenization buffer (1 ml/100 mg tissue). After centrifugation (8,000 x g, 10 minutes), supernatant was transferred to a clean tube. Determination of 8-isoprostane levels was made after alkaline hydrolysis of plasma and tissue-extracted supernatant. 8-iso-PGF2α is the main biologically active isoprostane produced by non-enzymatic peroxidation of arachidonic acid in membrane phospholipids. It circulates in plasma in two distinct forms, esterified phospholipids and as the free acid, in a ratio of approximately 2:1. Determination of 8-iso-PGF2α levels was done using 8-isoprostrane EIA kit (Cayman Chemical, Ann Arbor, MI) according to the manufacturer’s instructions.

### NOx levels determination

Prior to NOx levels determination, urine was diluted in assay buffer (1:10). Plasma samples were ultrafiltered using a 10 kDa molecular weight cut-off filter. Nitrate and nitrate are the final products of produced NO and measurement of total nitrate/nitrate is the best index of total NO production. The Nitrate/Nitrite assay kit (Cayman Chem, Ann Arbor, MI) is performed by a two step process, first converting nitrate to nitrite and second using the Griess reagent that converts nitrite to a deep purple azo compound, which is measured colorimetrically. NOx levels determination was done according to the manufacturer’s instructions.

### Western blot analysis

The CC muscle strips were homogenized in a lysing buffer containing 40 mmol/L HEPES, 1% Triton X-100, 10% glycerol, 1 mmol/L Na_3_VO_4_ and 1 mmol/L phenylmethylsulfonyl fluoride. The tissue lysate was centrifuged at 10,000 *g* and the supernatant was collected. The protein concentration was determined using a BCA protein assay kit. An aliquot of 40 μg protein from each sample was loaded per lane and resolved by SDS-polyacrylamide gel electrophoresis (SDS-PAGE) under reducing conditions. Proteins were subsequently transferred onto nitrocellulose membranes (BioRad, Hercules, U.S.A.). Membranes were blocked by treatment with 5% milk in Tris-buffered saline containing 0.05% tween 20, and probed with antibodies against the protein of interest as follow: eNOS (1:1000), nNOS (1:4000), Cu/Zn SOD (1:1000), Mn SOD (1:1000), RhoA (1:200), Rho-kinase α (1:500), Rho-kinase β (1:200), RhoGDI (1:2000), PDZ-RhoGEF (1:500), p115RhoGEF (1:200), p190RhoGAP (1:200), LARG (1:200), MYPT-1 (1:1000) and pMYPT-1 (1:1000). Next, membrane was incubated with a horseradish peroxidase-conjugated secondary antibody. Immunoreactivity was detected by enhanced chemiluminescence autoradiography and the protein expression was normalized to β-actin content.

### Corpora cavernosa membrane protein isolation

Briefly, CC tissues were pulverized, homogenized in lysis extraction buffer (100 mmol/L Tris−HCl, 1 mmol/L EDTA and 1 mmol/L EGTA containing phenylmethylsulfonyl fluoride (PMSF), protease inhibitor and phosphatase inhibitors), and centrifuged at 100.000*g* for 20 min at 4°C. Supernatant was collected as cytosolic fraction, and pellet was suspended in extraction buffer containing 1% Triton X-100 to obtain the membrane fraction. Protein was estimated using a commercially available kit from Bio Rad (Hercules, CA), and equal amounts of protein were loaded for Western blot, performed as described above.

### Drugs and solutions

Acetylcholine, atropine, Nω-Nitro-L-arginine methyl ester hydrochloride (L-NAME), PE and Y-27632 were purchased from Sigma Chemical Co. (St. Louis, U.S.A.). The antibodies against RhoA, Rho-kinase α and β isoforms, MYPT1 and p-MYPT1 were obtained from BD Biosciences (San Diego, CA, U.S.A.). The antibodies against RhoGAP, RhoGDI, RhoGEFs, were obtained from Santa Cruz Biotechnology (Santa Cruz, CA, U.S.A.). The antibodies against sGC α and β, PDE5, nNOS, CuZn SOD, Mn SOD and HuR were obtained from Millipore (Billerica, MA, U.S.A.). All other reagents used were of analytical grade. Stock solutions were prepared in deionized water and stored in aliquots at -20°C; dilutions were made up immediately before use.

### Data analysis

Relaxation responses are expressed as percentage of PE-induced maximum contraction. Curves were fitted to all the data using non-linear regression and half-maximum response (pEC_50_) to each drug expressed as–log molar (mol/L) was used to compare potency. All data were expressed as means ± S.E.M. of *n* experiments. The statistical significance of all differences between mean values was calculated using one-way ANOVA followed by Bonferroni’s *post hoc* test. (GraphPAD Software, version 6.00, San Diego, U.S.A.) Pearson correlation analysis was used to establish the relationship between the Rho-kinase signaling pathway and oxidative status while Forward Stepwise Regression was applied to predict the relationship between SOD expression in CC and predictor variables (IBM SPSS Statistics for Windows (version 20.0); IBM Corp., IBM Analytics, U.S.A). To reject the null hypothesis a level of *p*≤0.05 was considered to be statistically significant.

## Results

### RhoA/Rho-kinase pathway in the relaxation of the corpus cavernosum of diabetic mice

**Fig 1:**
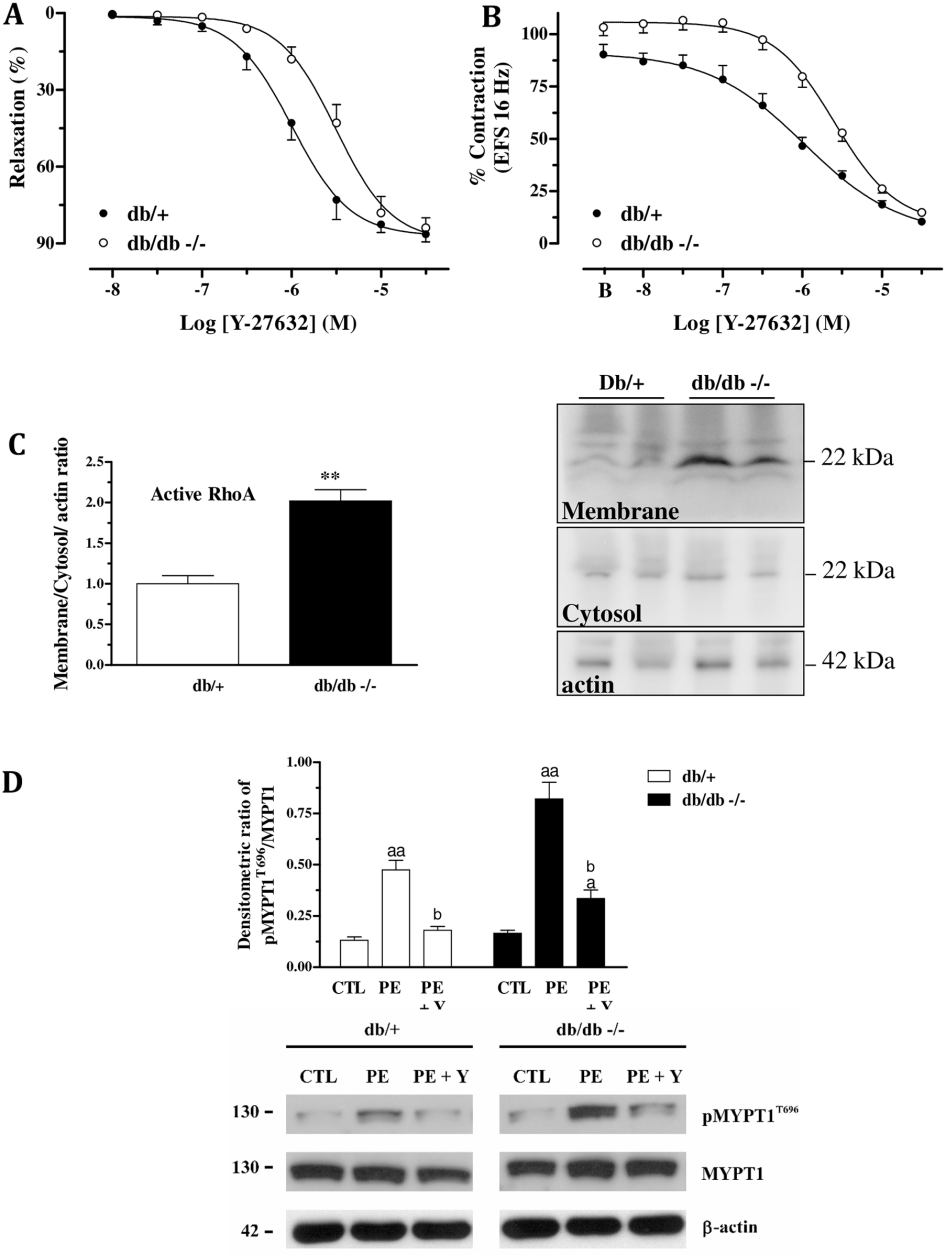
A) Relaxation response induced by the Rho-kinase inhibitor, Y-27632, in cavernosal tissue of db/+ and db/db mice pre-contracted with phenylephrine (PE 10 μmol/l); B) Inhibition of the electrical field stimulation-induced contraction in the absence (B) or in the presence of increasing concentrations of Y-27632 (EFS 16 Hz; Y-27632 0.01–30 μmol/l). C) Protein expression of RhoA in the corpora cavernosa of db/+ and db/db mice. Increased levels of active RhoA (membrane fraction) were observed in db/db compared db/+ mice. Densitometry values were normalized to β-actin and expressed as the fold increase of control db/+ mice. D) Phenylephrine (PE; 10 μmol/L) induced phosphorylation of MYPT-1. It was observed a higher magnitude of phosphorylation in db/db mice, compared to the db/+ mice. In the presence of Y-27632, MYPT-1 phosporylation was quite inhibited in db/+ mice and only partially inhibited in db/db mice. All data represents the mean ± S.E.M. of 4–6 experiments per group. *P<0.05 and **P<0.01, compared to db/+ mice; ^**a**^P< 0.05 compared to the respective control; ^**aa**^ P< 0.01 compared to the respective control; ^b^P< 0.05, compared to PE only.

Concentration-response curves for the Rho-kinase inhibitor Y-27632 (0.01–30 μM; n = 6) were obtained in CC strips of lean and diabetic mice. The potency (pEC_50_) of the relaxation induced by Y-27632 was significantly decreased in diabetic mice (pEC_50_: 5.51 ± 0.05) compared to lean mice (pEC_50_: 5.99 ± 0.02). No significant differences were seen in maximal responses (Fig 1A). In addition, EFS (16 Hz, in the presence of L-NAME and atropine) caused contraction of the CC which in basal conditions (represented on the graph by B in the x axis), was similar between the strains. Addition of cumulative concentrations of Y-27632 reduced (in a concentration-dependent manner) the magnitude of EFS-induced contraction. However, the magnitude of the inhibition produced by Y-27632 was reduced in db/db mice (pEC_50_: 5.59 ± 0.04) in comparison to db/+ mice (pEC_50_: 5.98 ± 0.05; Fig 1B).

Therefore, we evaluated membrane/cytosolic expression ratio of RhoA in the corpora cavernosa, which is an index of its activity. An increase in the membrane fraction of RhoA of db/db mice was observed, as shown in Fig 1C. Further, we measured the levels of phosphorylated MYPT-1, which is the target of Rho-kinase. MYPT-1 phosporylation was induced by the addition of phenylephrine (10 μmol/L) to the corpus cavernosum. Phenylephrine caused an increase in the levels of pMYPT-1 in both strains. However, this increase was significantly higher in the corpus cavernosum of db/db mice. Addition of the Rho-kinase inhibitor Y-27632 totally prevented the phosporylation of MYPT-1 in db/+ mice, while it was only partially diminished in the corpus cavernosum of db/db mice (Fig 1D).

**Fig 2:**
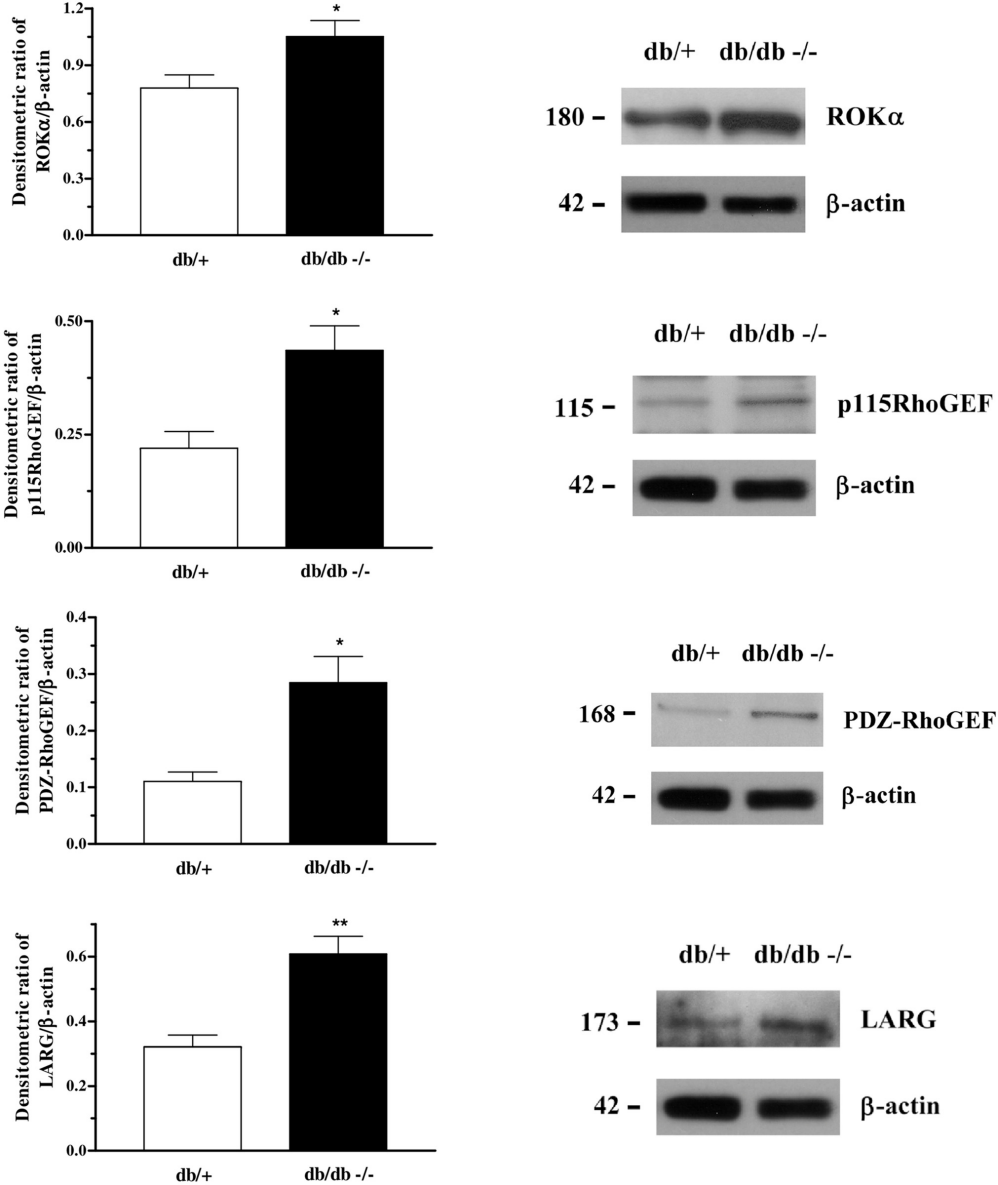
Increased protein expression of Rho-kinase α and RhoGEFs subunits p115RhoGEF, PDZ-RhoGEF and LARG in the corpora cavernosa of db/db mice. Data represents the mean ± S.E.M. of 4–6 experiments per group. *P<0.05 and **P<0.01, compared to db/+ mice.

Further, we measured the expression of proteins and enzymes of the Rho-kinase pathway in the corpus cavernosum of db/+ and db/db mice. No differences were seen in the expression of RhoA, RhoGDI, ROKβ and p190Rho-GEF between the strains (data not shown). However, increased expression of ROKα, p115Rho-GEF, PDZ-RhoGEF and LARG was observed in the corpus cavernosum of db/db mice (Fig 2).

### NO/sGC/cGMP pathway in the relaxation of the corpus cavernosum of diabetic rats

KCl (80 mmol/L)-induced contractions were not significantly different in CC between strains (db/+: 2.5 ± 0.2 mN, db/db: 2.0 ± 0.3 mN, n = 6 per group). Isolated cavernosal segments were contracted with PE (10 μmol/L), which achieved 70–80% of KCl-induced maximum contraction. There was no significant difference in PE-induced contraction between strains (data not shown).

Endothelium integrity was assessed by a single concentration of acetylcholine (10 μmol/L). A lower relaxation induced by 10 μmol/L of acetylcholine was observed in db/db mice (data not shown).

**Fig 3:**
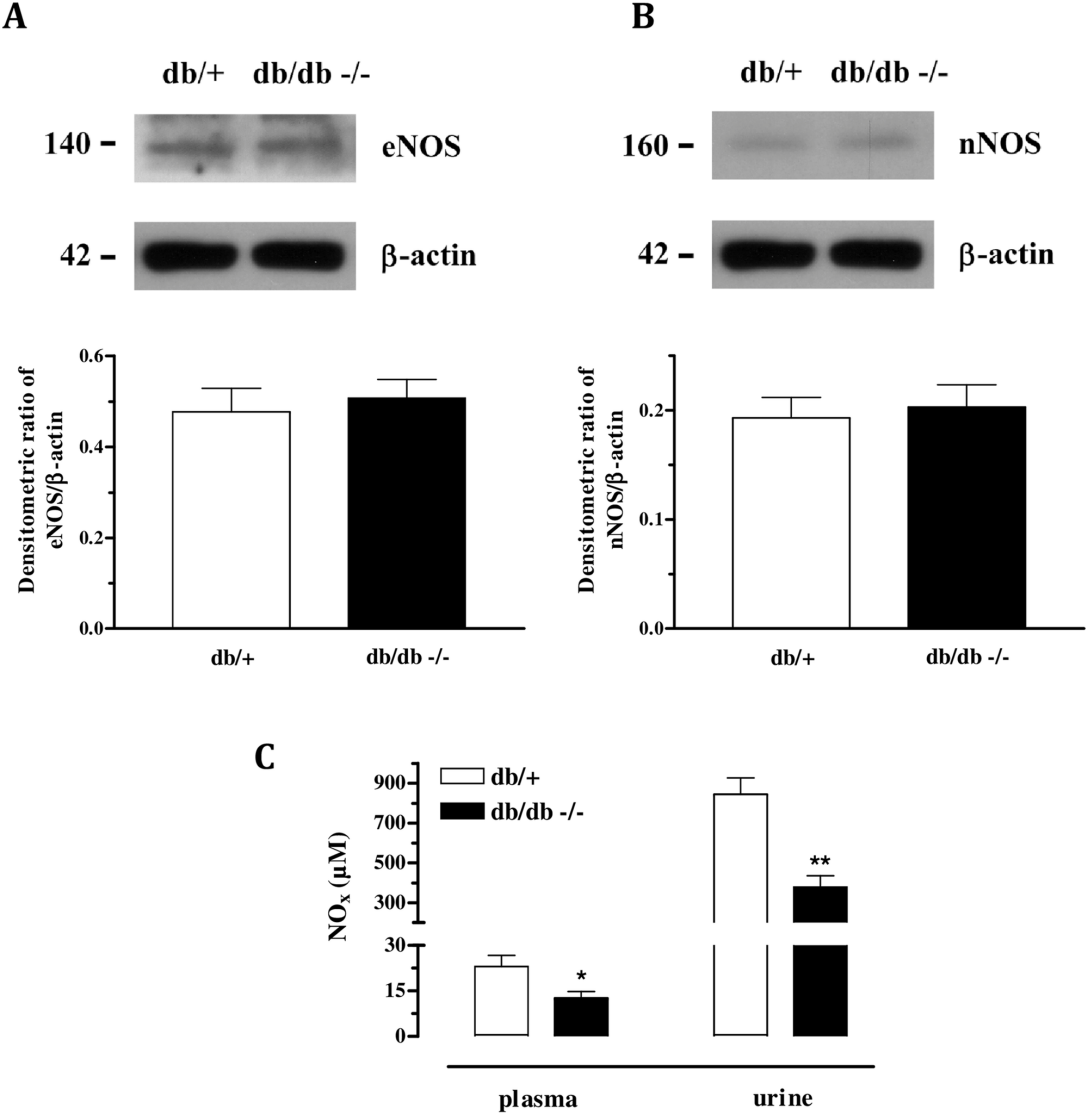
Protein expression of eNOS (Panel A) and nNOS (panel B) were similar in the CC of db/+ and db/db mice. Panel C shows decreased plasma and urine levels of nitrite/nitrate (NOx) in db/db mice compared to db/+. Data represents the mean ± S.E.M. of 4–6 experiments. *P<0.05 and **P<0.01, compared to the db/+ mice.

**Fig 4:**
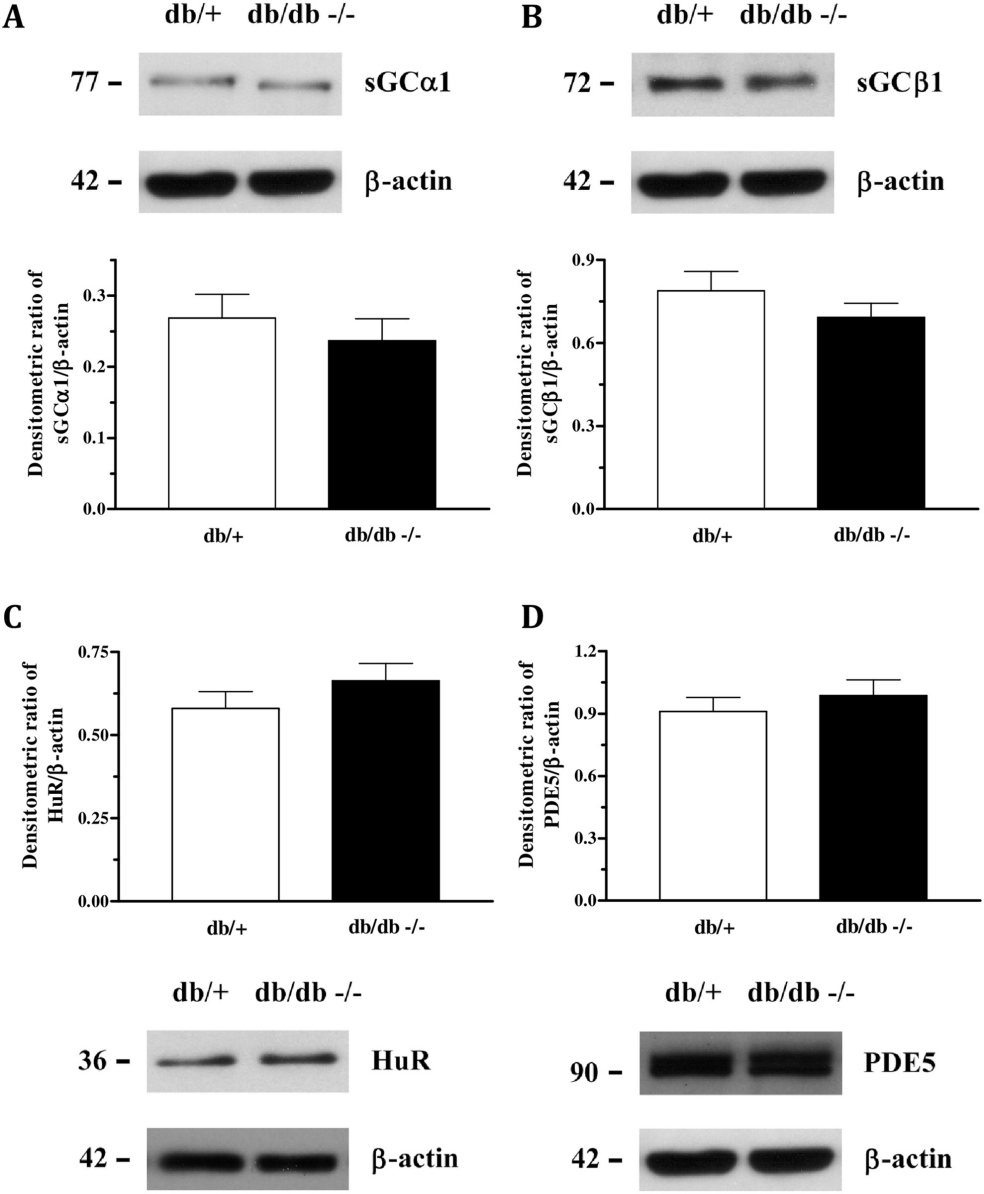
Protein expression of sGCα (Panel A), sGCβ (panel B), HuR (Panel C) and PDE5 (Panel D) were similar in the CC of db/+ and db/db mice. Data represents the mean ± S.E.M. of 4–6 experiments.

The expression of either neuronal or endothelial nitric oxide synthase was not different between the groups (Fig 3A and 3B). Nevertheless, NOx levels were significantly diminished in plasma and urine of diabetic mice (Fig 3C). Additionally, the expression of both subunits of sGC (Fig 4A and 4B), HuR and PDE5 (Fig 4C and 4D) was similar between the strains.

### Lipid peroxidation evaluation

**Fig 5:**
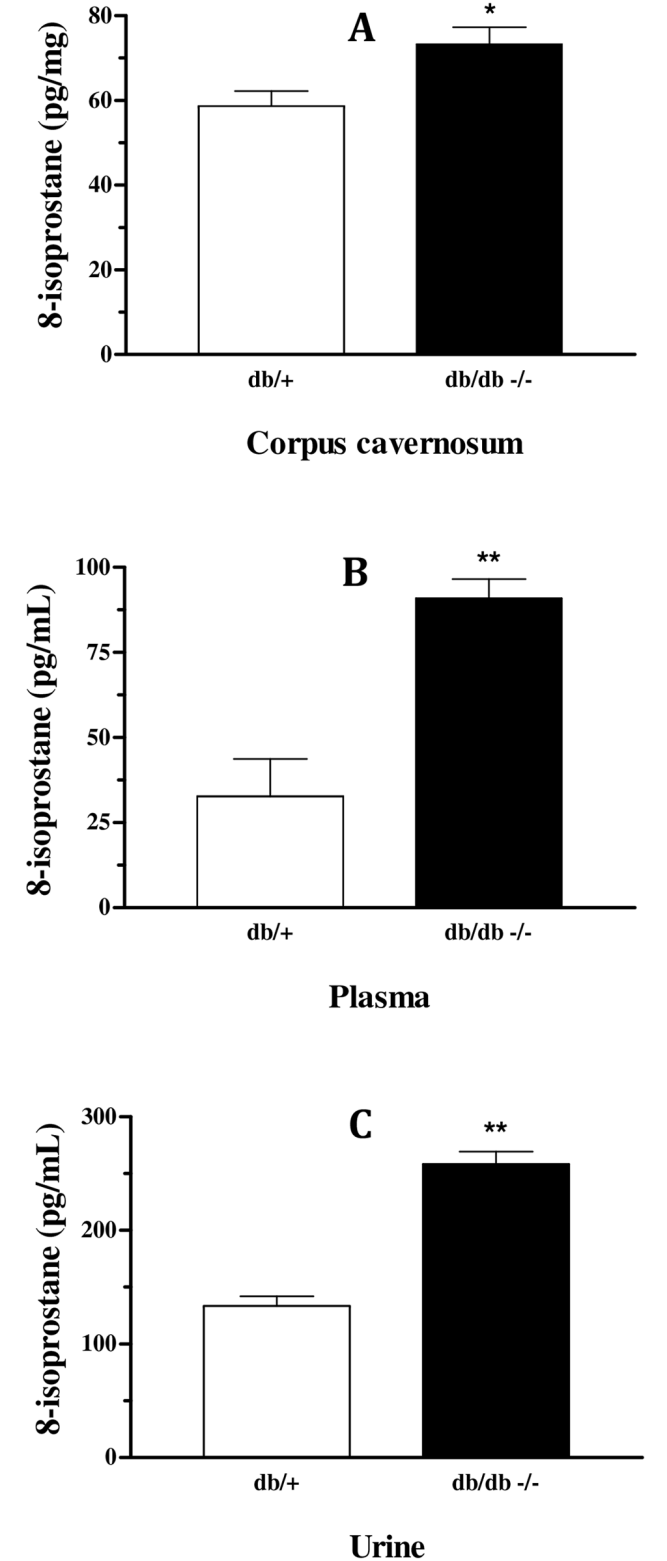
Increased lipid peroxidation, measured by formation of 8-isoprostane, in CC (Panel A), plasma (Panel B) and urine (Panel C) of db/db mice compared to db/+. Data represents the mean ± S.E.M. of 4–6 experiments *P<0.05 and **P<0.01, compared to db/+ mice.

Next, we evaluated lipid peroxidation by measuring 8-isoprostane levels. In db/db mice, increased levels of 8-isoprostane were observed in corpus cavernosum (db/+: 59 ± 4; db/db: 73 ± 4 pg/mg; Fig 5A), plasma (db/+:33 ± 11; db/db: 91 ± 6 pg/ml; Fig 5B) and urine (db/+:133 ± 9; db/db: 258 ± 11 pg/ml; Fig 5C).

### SOD activity and expression evaluation

**Fig 6:**
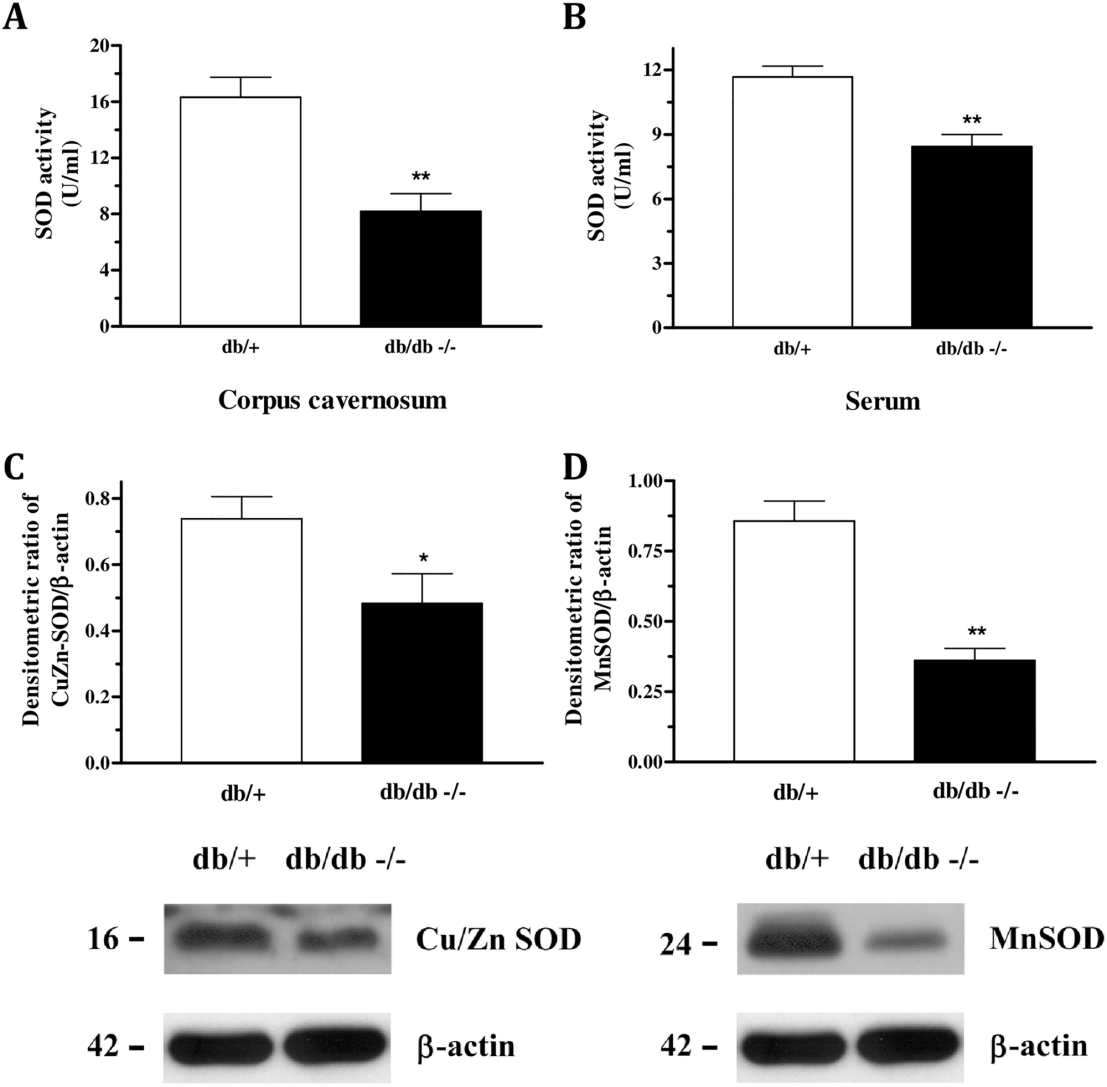
Decreased activity of the antioxidant enzyme SOD in the corpus cavernosum (Panel A) and serum (Panel B) of db/db mice compared to db/+ mice. Protein expression of CuZn (Panel C) and Mn SOD (Panel D) measured by western blot were diminished in the CC of db/db mice. Data represents the mean ± S.E.M. of 4–6 experiments. *P<0.05 and **P<0.01, compared to db/+ mice.

SOD activity was measured in either corpus cavernosum or serum while protein expression of the Cu/Zn SOD and Mn SOD was measured only in the corpora cavernosa. In db/db mice, it was observed decreased activity of total SOD (panel 6A and 6B) and expression of both subunits (Fig 6C and 6D).

### Correlation between oxidative status and Rho-kinase pathway

Pearson correlation and forward stepwise Regression were performed in order to evaluate the interaction between oxidative status and Rho-kinase pathway. It was observed several associations among variables of the study, suggesting a cross-talk between these pathways. There was a marked negative correlation between ROCKα and SOD activity on serum (S1 Fig). Further, regression test showed that the db/db strain is the determinant factor of the diminished expression of SOD in the CC (S2 Fig). These data and statistical analysis were shown in supporting information file.

## Discussion

In this study, we tested the hypothesis that impaired corpus cavernosum reactivity induced by the Rho-kinase inhibitor might be accompanied by upregulation of the Rho-kinase signalling pathway, decreased NO bioavailability and increased oxidative stress in db/db mice. First, we evaluated the relaxation induced by the Rho-kinase inhibitor, Y-27632. In corpus cavernosum of db/db mice, we observed decreased potency in the relaxing response to Y-27632. Similarly, the inhibition produced by Y-27632 on the EFS-induced contraction was right-shifted in this strain, which is suggestive of an upregulation of the expression and/or activity of Rho-kinase and its regulatory proteins. Thus, we further evaluated the activity and expression of proteins involved in the RhoA/Rho-kinase signalling pathway. Activation of RhoA begins with activation by a contractile stimulus via G-protein coupled receptors. Inactive RhoA is bound to RhoGDI (Rho-guanine dissociation inhibitor) in the cytosol. RhoA activation is catalyzed by guanine nucleotide exchange factors (Rho-GEFs) and induces the dissociation of RhoA from RhoGDI. Next, RhoA translocates from the cytosol to the membrane, which, in turn, activates the Rho-kinase. Rho-kinase inhibits myosin light chain phosphatase activity by phosphorylating the regulatory subunit MYPT-1, and it enhances the contractile response. [23, 24] Therefore, we measured the expression of membrane and cytosolic fractions of RhoA, as an index of its activity, as previously described. [25–27] Membrane fraction of RhoA was increased in db/db mice, indicating that RhoA is more active in this strain. This finding corroborates the increased RhoA translocation to the membrane in streptozotocin-induced diabetic mice. [27] We further measured MYPT-1 phosphorylation induced by adrenergic stimulation. Erectile dysfunction associated to age, streptozotocin-induced diabetes and heart failure were shown to exhibit increased basal levels of phosphorylated MYPT-1. [25, 27, 28] Unexpectedly, herein we found similar basal levels of pMYPT-1 between the strains. It is possible that the phosphatase which dephosphorylates MYPT-1 might be more active as a compensatory mechanism, keeping unchanged the basal levels of pMYPT-1. On the other hand, phenylephrine induced MYPT-1 phosphorylation in both strains, however, the magnitude of phosphorylation was higher in db/db mice, corroborating the idea of increased activity of Rho-kinase in this strain. In addition, the Rho-kinase inhibitor, Y-27632, was less effective to prevent MYPT-1 phosphorylation in db/db mice. It suggests that both RhoA and Rho-kinase are more active in db/db mice. Further, expression of Rho-kinase α and RhoGEFs subunits (p115RhoGEF, pdzRhoGEF and LARG) were increased in db/db mice. Together, these data demonstrate the upregulation of the RhoA/Rho-kinase pathway in db/db mice. These results are in agreement with others showing that this pathway is increased in several vascular disorders including erectile dysfunction. [8, 29]

Additionally, we have previously shown that basal release of endothelial nitric oxide is necessary to maintain the basal tonus of the corpus cavernosum, since in eNOS-/- mice there is an overexpression of the Rho-kinase-associated proteins, favouring the flaccid state of the penis and contributing to erectile dysfunction. [21] Further, we have recently shown a significant reduction in the relaxation induced by ACh and EFS (but not the NO donor sodium nitroprusside—SNP) in db/db mice compared to the db/+ strain, showing that the NO/sGC/cGMP pathway is impaired in db/db. [22] Other previous studies have shown similar results in db/db mice and type-1 model of diabetes. [13, 30] To assess the NO/sGC/cGMP pathway herein, we measured the protein expression of endothelial and neuronal NO synthase, sGC (α and β subunits), HuR (the sGC mRNA-stabilizing protein) and PDE5. Expression of the sGC, HuR and PDE5 proteins were similar in both strains, suggesting that the downstream signalling pathway of NO is preserved in db/db mice. It corroborates our recent study showing that BAY 41-2272- (sGC stimulator) and SNP- induced relaxation was similar in db/+ and db/db mice. [22] In addition, in the present study, both eNOS and nNOS expression were demonstrated to be similar between the strains. Nonetheless, NOx levels in plasma and urine were shown to be reduced in db/db mice, suggesting decreased NO bioavailability in this strain.

In our previous study, we showed impaired relaxation stimulated by ACh and EFS in db/db mice, which corroborates the reduced levels of NOx. [22] Other previous study showed reduced relaxation induced by ACh and EFS but not by SNP in high fat diet obese mice. [31] Taken together, the expression of eNOS, nNOS, sGC, HuR and PDE5 as well as the NOx levels suggest that although protein expression was not changed, NO bioavailability was reduced and might be responsible via Rho-kinase pathway upregulation. A previous study reported erectile dysfunction in obese Zucker rats compared to lean Zucker rats with no differences in the expression of nNOS in the penile arteries in either strains. However, erectile dysfunction was associated with nNOS uncoupling due to increased oxidative stress. [17] In non-obese type II diabetic rats ED was not associated to nNOS expression changes. Nevertheless, in opposition to our findings, this study reported that erectile dysfunction was associated with decreased eNOS expression and phosphorylation at Ser^1177^. [32] In addition, it is well known that db/db mice is highly insulin resistant and insulin seems to have a modulatory effect on the NOS activity. A recent study showed that in a model of insulin resistant rats, expression and activity of NOS as well as NOx levels in paraventricular nucleus were significantly reduced. [33] On the other hand, another study showed that in young spontaneously hypertensive rats, treatment with insulin sensitizer pioglitazone does not change NOS expression and activity but improves SOD activity. [34] Together, these data suggests that insulin resistance might be responsible by decreased NO bioavailability, by either changing NOS or ROS. In Zucker rats with type 2 diabetes mellitus, treatment with low dose of pioglitazone prevents erectile dysfunction prior to changes in the glucose levels, which suggests that the effects of PPAR-γ activation might be beyond of insulin resistance improvement. [35]. On the other hand, in mice fed with high fat diet, treatment with metformin restored insulin resistance and prevented erectile dysfunction. [36] However, more studies are necessary to elucidate the role of insulin resistance in erectile dysfunction.

Several studies have reported the relationship between ROS and ED in disorders such as arterial hypertension, atherosclerosis, hypercholesterolemia, diabetes, sleep apnea, aging and cigarette. [10–18] Herein, although NOS expression was not altered in db/db mice, decreased NOx levels suggest diminished NO bioavailability and it might be caused by increased oxidative stress. We have previously shown increased formation of superoxide anion, which was accompanied by increased expression of NADPH oxidase subunits and decreased total antioxidant status in db/db strain [22]. Here, we found diminished expression and activity of the antioxidant enzyme SOD in db/db mice, which corroborates to the idea of increased superoxide anion formation. Excess of superoxide anion production might be responsible by scavenge NO to form peroxynitrite (ONOO^-^) [37] which may cause eNOS uncoupling [38] or lipid peroxidation of cell membrane [39]. Indeed, we found increased levels of 8-isoprostane in tissue and fluids of db/db mice. Measurement of 8-isoprostane was taken as an index of lipid peroxidation, since 8-isoprostane is produced by non-enzymatic peroxidation of arachidonic acid in membrane phospholipids. These findings are in agreement with previous studies showing decreased SOD activity and increased lipid peroxidation in the CC of diabetic rats as well as in hypertensive rats. [40, 41] When risk factors for cardiovascular disease are present, the enhanced oxidative stress, demonstrated by higher expression of NADPH oxidase, increased superoxide anion formation and eNOS uncoupling, might be the leading cause of erectile dysfunction. [42, 43] Altogether, these data support the idea that even with normal expression of NOS in the corpus cavernosum of db/db mice, the lower levels and activity of the antioxidant enzymes SOD contributes to increase superoxide anion levels, which decreases NO bioavailability, increasing peroxynitrite levels, causing eNOS uncoupling and tissue damage by lipid peroxidation which impairs corpus cavernosum relaxation and lead to erectile dysfunction.

In addition, the association test suggested a cross-talk among variables in the RhoA/ROCK signaling pathway and oxidative status. There was a marked negative correlation between ROCKα expression and SOD activity on serum. The experimental model (db/db) seems to be the determinant factor of the reduced expression of SOD in the CC. In summary, our data suggest that db/db mice strain leads to decreased SOD expressed in the CC, which is correlated to increased lipid peroxidation, decreased NOx levels and SOD activity on serum. This last one is strongly correlated to the enhanced ROCKα expression in CC and consequently, amplification of the Rho-kinase signaling pathway. These data corroborate the previous finding that in rat aorta ROS activates the RhoA/Rho-kinase pathway. [26]

In conclusion, our data demonstrate that in db/db mice, upregulation of the RhoA/Rho-kinase signalling pathway was accompanied by decreased NO bioavailability and increased oxidative stress which might be responsible by impaired relaxation of the corpus cavermosum of db/db mice.

## Supporting Information

S1 Fig Schematic representation of the correlation between RhoA/ROCK signaling pathway and oxidative status in db/db mice. Values are described below and only comparison exhibiting significant correlation are shown. Pearson Correlation: ^#^p≤ 0.05 = significance; *p≤ 0.01 = significance.*RhoA and ROCKα p = 0.00, r = 0.881; ^#^RhoA and Rockβ p = 0.02, r = 0.784); *ROCKα and p115GEF p = 0.00, r = 0.857; *ROCKα and pdzGEF p = 0.00, r = 0.850; *ROCKα and SOD serum p = 0.00, r = −0.853; ^#^SOD serum and SODCC p = 0.01, r = 0.829; *SOD serum and pdzGEF p = 0.00, r = −0.888; *SOD serum and p115GEF p = 0.00, r = −0.937; *SOD serum and NOx plasma p = 0.00, r = 0.886; ^#^NOx plasma and 8-isoprostane p = 0.01, r = −0.837; *p115GEF and pdzGEF p = 0.01, r = 0.933; ^#^pdzGEF and SODCC p = 0.04, r = −0.813; ^#^SOD CC and 8-isoprostane p = 0.05, r = −0.812.
(DOCX)

S2 Fig Evaluation of the target SOD expression in the corpus cavernosum (SODCC) according to the predictors included in the model. Data revealed that the strain db/db is the predictor of the reduced amount of SOD expressed in the corpus cavernosum.
(DOCX)
