# Supplementary material for: Impaired Corpus Cavernosum Relaxation Is Accompanied by Increased Oxidative Stress and Up-Regulation of the Rho-Kinase Pathway in Diabetic (Db/Db) Mice
Source: PLoS One. 2016 May 26;11(5):e0156030. doi: 10.1371/journal.pone.0156030 (PMC4882003; doi:10.1371/journal.pone.0156030)
Supplement: S1 Fig — Values are described below and only comparison exhibiting significant correlation are shown. Pearson Correlation: #p≤ 0.05 = significance; *p≤ 0.01 = significance.*RhoA and ROCKα p = 0.00, r = 0.881; #RhoA and Rockβ p = 0.02, r = 0.784); *ROCKα and p115GEF p = 0.00, r = 0.857; *ROCKα and pdzGEF p = 0.00, r = 0.850; *ROCKα and SOD serum p = 0.00, r = −0.853; #SOD serum and SODCC p = 0.01, r = 0.829; *SOD serum and pdzGEF p = 0.00, r = −0.888; *SOD serum and p115GEF p = 0.00, r = −0.937; *SOD serum and NOx plasma p = 0.00, r = 0.886; #NOx plasma and 8-isoprostane p = 0.01, r = −0.837; *p115GEF and pdzGEF p = 0.01, r = 0.933; #pdzGEF and SODCC p = 0.04, r = −0.813; #SOD CC and 8-isoprostane p = 0.05, r = −0.812. (DOCX) [file pone.0156030.s001.docx]

**Pearson Correlation Analysis**


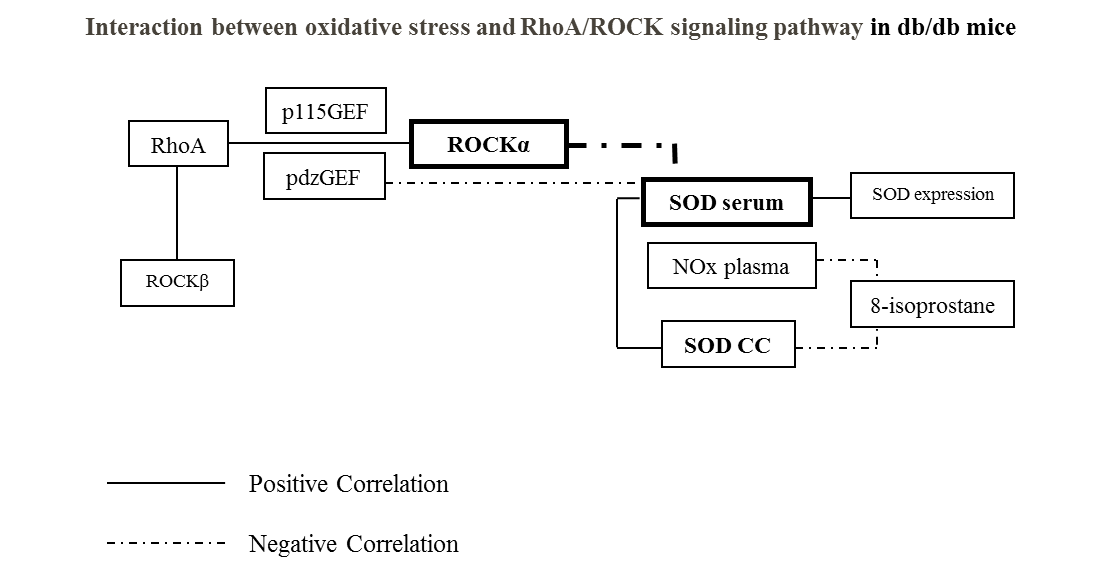
It was observed several associations among variables of the study in the RhoA/Rock signaling pathway and oxidative status (Pearson correlation), as shown in S1 Fig, which suggests a cross-talk between these pathways. There was a marked negative correlation between ROCKα expression and SOD activity on serum.

**S1 Fig.** Schematic representation of the correlation between RhoA/ROCK signaling pathway and oxidative status in db/db mice. Values are described below and only comparison exhibiting significant correlation are shown.

Pearson Correlation: ^#^p≤ 0.05 = significance; *p≤ 0.01 = significance

*RhoA and ROCKα p = 0.00, r = 0.881;

^#^RhoA and Rockβ p = 0.02, r = 0.784);

*ROCKα and p115GEF p = 0.00, r = 0.857;

*ROCKα and pdzGEF p = 0.00, r = 0.850;

*ROCKα and SOD serum p = 0.00, r = - 0.853;

^#^SOD serum and SODCC p = 0.01, r = 0.829;

*SOD serum and pdzGEF p = 0.00, r = - 0.888;

*SOD serum and p115GEF p = 0.00, r = - 0.937;

*SOD serum and NOx plasma p = 0.00, r = 0.886;

^#^NOx plasma and 8-isoprostane p = 0.01, r = - 0.837;

*p115GEF and pdzGEF p = 0.01, r = 0.933;

^#^pdzGEF and SODCC p = 0.04, r = - 0.813;

^#^SOD CC and 8-isoprostane p = 0.05, r = - 0.812
