# Supplementary material for: Impaired Corpus Cavernosum Relaxation Is Accompanied by Increased Oxidative Stress and Up-Regulation of the Rho-Kinase Pathway in Diabetic (Db/Db) Mice
Source: PLoS One. 2016 May 26;11(5):e0156030. doi: 10.1371/journal.pone.0156030 (PMC4882003; doi:10.1371/journal.pone.0156030)
Supplement: S2 Fig — Data revealed that the strain db/db is the predictor of the reduced amount of SOD expressed in the corpus cavernosum. (DOCX) [file pone.0156030.s002.docx]

**Forward Stepwise Regression Analysis**

Using Forward Stepwise Regression, that predict continuous target based on relationship between the target (SOD expression in CC; SODCC) and one or more predictors (all variables were tested) it was observed that the experimental model (db/db strain) was the determinant factor of the diminished expression of SOD in the CC (S2 Fig).


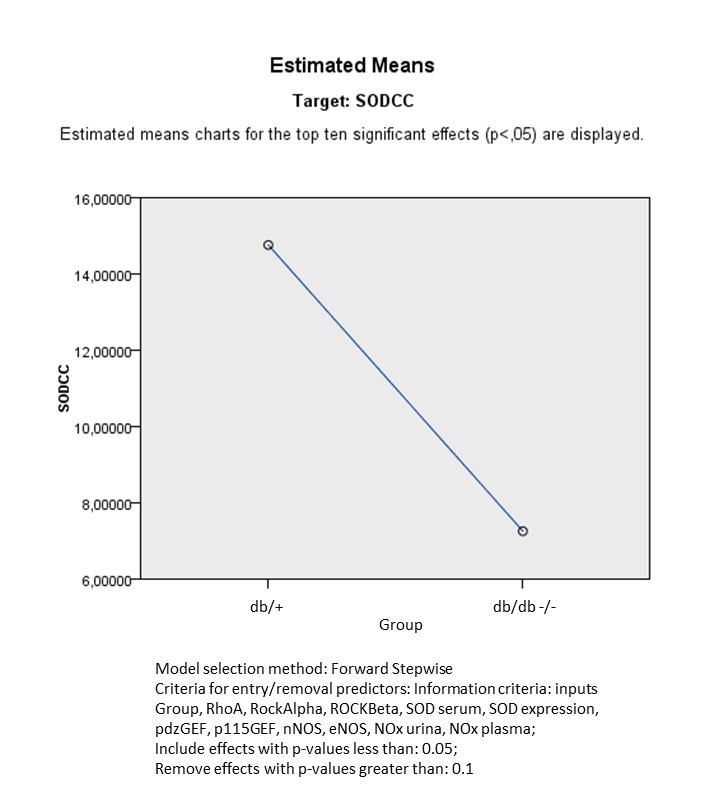


**S2 Fig.** Evaluation of the target SOD expression in the corpus cavernosum (SODCC) according to the predictors included in the model. Data revealed that the strain db/db is the predictor of the reduced amount of SOD expressed in the corpus cavernosum.
